# A pilot randomised controlled trial investigating a mindfulness-based stress reduction (MBSR) intervention in individuals with pulmonary arterial hypertension (PAH): the PATHWAYS study

**DOI:** 10.1186/s40814-018-0270-z

**Published:** 2018-05-21

**Authors:** R. M. R. Tulloh, V. Garratt, J. Tagney, J. Turner-Cobb, E. Marques, R. Greenwood, L. Howard, W. Gin-Sing, A. Barton, P. Ewings, P. Craggs, W. Hollingworth

**Affiliations:** 1 Department of Congenital Heart Disease, Bristol Heart Institute, University Hospitals Bristol NHS Foundation Trust, Upper Maudlin Street, Bristol, BS2 8BJ UK; 2 Department of Psychology, Bournemouth University, Poole, BH12 5BB UK; 3 Health Economics, Musculoskeletal Research Unit, Southmead Hospital, University of Bristol, Bristol, BS10 5NB UK; 4 Research Design Service, Taunton and Somerset Hospital, Taunton and Somerset NHS Foundation Trust, Taunton, TA1 5DA UK; 5 Bristol Heart Institute, Upper Maudlin Street, Bristol, BS2 8HW UK; 6 Department Congenital Heart Disease, Bristol Royal Hospital for Children, Upper Maudlin Street, Bristol, BS2 8BJ UK; 7 ITTC Building, Plymouth Science Park, Plymouth University School of Medicine and Dentistry, Plymouth, PL6 8BX UK; 8 Hammersmith Hospital, Du Cane Road, London, UK; 9 Research Design Service, University Hospitals Bristol NHS Foundation Trust, Upper Maudlin Street, Bristol, BS2 8BJ UK; 10 School of Social and Community Medicine, University of Bristol, Canynge Hall, 39 Whatley Road, Bristol, BS8 2PS UK

**Keywords:** Pulmonary arterial hypertension, Mindfulness-based stress reduction, Psychology, Economic evaluation

## Abstract

**Background:**

Pulmonary arterial hypertension (PAH) is an uncommon condition with progressive heart failure and premature death. Treatment costs up to £120,000 per patient per year, and the psychological burden of PAH is substantial. Mindfulness-based stress reduction (MBSR) is an intervention with the potential to reduce this burden, but to date, it has not been applied to people with pulmonary hypertension. We wished to determine whether a trial of MBSR for people with PAH would be feasible.

**Methods:**

A customised gentle MBSR programme of eight sessions was developed for people with physical disability due to PAH, and they were randomised to group-based MBSR or treatment as usual. The completeness of outcome measures including Beck Anxiety Index, Beck Depression Inventory and standard physical assessment at 3 months after randomisation were recorded. Health care utilisation was measured. Attendance at the sessions and the costs involved in delivering the intervention were assessed. Semi-structured interviews were conducted to explore the acceptability of the MBSR intervention and when appropriate the reasons for trial non-participation.

**Results:**

Fifty-two patients were recruited, but only 34 were randomised due to patients finding it difficult to travel to sessions. Twenty-two completed all questionnaires and attended all clinics, both routine and additional in order to collect outcomes measures. The MSBR sessions were delivered in Bristol, Cardiff and London, costing, on average, between £2234 (Cardiff) and £4128 (London) per patient to deliver. Attendance at each session averaged between two patients in Bristol and Cardiff and three in London. For those receiving treatment as usual, clinician blinding was achievable. Interviews revealed that people who attended MBSR found it interesting and helpful in managing their symptoms and minimising the psychological component of their disease.

**Conclusions:**

We found that attendance at group MBSR was poor in people with chronic PAH within the context of a trial. Achieving better MBSR intervention attendance or use of an Internet-based programme might maximise the benefit of MBSR.

## Background

Pulmonary arterial hypertension (PAH) is an unpredictable and life-limiting condition. It results in progressive increases in pulmonary vascular resistance, ultimately leading to right ventricle failure and premature death [1]. It is characterised by debilitating progressive symptoms of severe breathlessness, fatigue, cyanosis and haemoptysis and declining functional ability. Precise national prevalence data are unconfirmed, but 5538 patients are monitored in the UK (Dr. L Howard personal communication from National Audit of PAH, 2010), and the number is rising with increasing awareness.

The causes of PAH range from those with idiopathic PAH (IPAH), with a short life expectancy of 2.8 years without treatment [2], to those associated with congenital heart disease (APAH), who commonly live into their fourth decade for whom treatment remains palliative [3]. Treatment can be invasive, painful and upsetting, and it is not clear whether new methods of treatment have increased life expectancy [3]. Most patients are seen in clinic at least twice a year and may require hospital admission to initiate treatment. Care costs are up to £120,000 per person per year [4]. Supporting patients as they cope with the psychological distress associated with PAH is an important adjunct of care. Living with an unpredictable future, complex treatments and lifestyle changes means that the emotional burden of PAH is substantial [5].

### Pulmonary hypertension and psychological health

The symptoms of PAH can have a devastating impact on daily life. Many people cannot work and have limited mobility and independence, and women are advised to avoid pregnancy [6]. They may have consistently high levels of anxiety (20%), depression (26%) and panic (25%) [7], associated with decreased quality of life and physical functioning [8], as well as an increase in concern about symptoms [5, 9, 10]. Worryingly, only 24.1% of patients reported seeking professional help for psychological effects [7]. Anxiety both exacerbates and mimics symptoms of PAH making it difficult for patients to interpret their symptoms and increasing their need for support. For example, breathlessness and chest pain are features of anxiety but also of PAH. Qualitative analysis from research with PAH patients revealed that they consciously ‘hold back’ in order to avoid provoking symptoms and increased anxiety due to a heightened sense of awareness as they approached each activity, for fear of not being able to catch their breath [11]. Anxiety may also stem from the uncertainties associated with prognosis, treatment and course of the illness [12].

### Mindfulness-based stress reduction intervention

Mindfulness is defined as ‘the awareness that emerges through paying attention on purpose, in the present moment, and non-judgmentally to the unfolding of experience moment by moment.’ [13]. Mindfulness-based stress reduction (MBSR) is a group-based course that runs for a recommended eight weekly sessions and includes education about stress and its effects on the body, becoming aware of unhelpful attitudes and thinking patterns, defining mindfulness and using it in everyday life, formal meditation techniques and gentle stretching. Participants are given a resource booklet to accompany the course and asked to continue practising what they have learnt at home. Benefits include increased ability to cope, improvements in mental health, physical functioning, well-being, quality of life, decreased psychological distress, enhanced functional status and reduced physical symptoms for people with chronic pain, cancer, heart disease, depression, anxiety, fibromyalgia, rheumatoid arthritis, type 2 diabetes and chronic fatigue [14–16]. Positive results have been demonstrated for continued practice and maintained effect after 6 months [17, 18]. Significant effects have been observed with eight (2.5-h) weekly sessions of MBSR on blood pressure [19], vascular disease [20], lifestyle stress, blood pressure [21] and general cardiovascular diseases [22]. To date, there has been no intervention for people with pulmonary hypertension.

### Aims and objectives

The overall aim of this study was to assess the feasibility and pilot the methods of a randomised controlled trial comparing MBSR with treatment as usual. This would examine the clinical and cost-effectiveness of an MBSR intervention for people with PAH aged over 16. The pilot study reported here mirrored as closely as possible the intended design and conduct of a larger trial, so that the information it provided could be used to inform the design of that trial.

The specific objectives of the pilot study were to:

1. Assess the processes of participant identification, recruitment, randomisation and collection of outcome data, including rates of recruitment, attrition, questionnaire completion and overall follow-up. Assess logistics, in particular how to deliver a group-based intervention in a number of geographical locations.
2. Assess acceptability to participants of the protocol, including receiving the intervention, being in the control group and remaining in the study for 15 months.
3. Develop and test data collection methods, including outcomes and resource use.
4. Obtain descriptive distributional data on the outcome measures for this population, including measures of spread, to inform sample size calculation.
5. Estimate the cost of delivering the MBSR intervention, exploring different settings and group size.

## Methods

### Participants

We included those patients with a clinical diagnosis of PAH who were aged 16 years or older (due to the need for participants to self-reflect and participate in a group of mixed ages) and who were deemed physically and psychologically stable enough to participate in a study for approximately 15 months by their care team. We excluded those with significant learning disabilities and who lacked capacity to consent or who were not fluent in English. In addition, we excluded those who were unable to take part (e.g. prior commitments, excess travel) or who were involved in simultaneous research projects.

We planned to randomise 42 participants into the pilot study in a 1:1 ratio. The group size was intended to be seven participants. We wished to have three groups in each of the randomised intervention or treatment as usual cohorts. This suggested a total of 42 participants and would allow for some dropout during the study. The research assistant approached potentially eligible participants with information about the trial following a routine clinic visit. Initial recruitment was at a single site, Bristol, which included patients from a wide catchment area, including South Wales and South West England. If they were interested in participating, full information about the trial was provided, and participants were given the opportunity to ask questions. If the participant wished to proceed, written informed consent was sought along with the completion of baseline questionnaires. To avoid lengthy travel times, intervention groups were arranged in various outlying locations. We aimed for a minimum of seven participants per intervention group, necessitating recruitment of at least 14 participants within a geographical area from which participants were happy to attend a central site to receive the intervention (if so randomised). MBSR sessions were offered in the early afternoon from 12 to 2 pm, throughout the year. Baseline measures were collected, and once there were sufficient patients, the intention was to randomise on a 1:1 basis to intervention or control.

It took 18 months to recruit 18 participants in the Bristol centre. It became apparent that it would not be possible to undertake randomisation as intended, due to potential participants living in widely dispersed areas making attendance at weekly group sessions difficult. We then over recruited (with ethics committee approval) patients to a waiting list of potential participants living near two geographic locations (Bristol and Cardiff) who were not randomised until the group size at that site became large enough. We also expanded the recruitment to an additional site. This additional site, Hammersmith Hospital, is a National Specialist Pulmonary Hypertension Centre that manages 1200 people with pulmonary hypertension. The majority of the patients lived within a 2-hour radius, but some travelled to the centre from across the UK. Only those close to London were included from this site for ease of travelling to the MBSR sessions. Due to low numbers, the randomisation ratio was also increased to ensure a viable number of patients could be recruited to form MBSR groups of sufficient size (up to a maximum of 3:1). Additionally, we randomised (with ethics committee approval) a smaller group of seven participants with the additional members of the MBSR group made up from patients from the same clinic but with different diagnoses.

Participants were informed of their allocation by letter along with details of venue and timings for the MBSR group sessions. After baseline (T1), follow-up was planned for 10–12 weeks (T2), 6 months (T3) and 15 months (T4) after randomisation. All patients were followed up to 6 months. However, as recruitment took much longer than originally planned, only patients (*n* = 18) from the Bristol centre were followed for the additional time point of 15 months post-randomisation. For this reason, in our analysis, we focussed on 6-month outcomes.

### Acceptability of the intervention

Adaptations to the MBSR programme were based on advice and material given by Michael Speca (personal communications 20 October 2010 to 22 February 2011) and the experiences of patients. Prior to the pilot study, we invited four patients to undergo baseline assessment, attend the 8-week intervention and have an assessment at follow-up. Based on this, we further tailored the MBSR programme to patients with PAH.

An experienced clinical psychologist and assistant psychologist delivered the course in a group setting in eight 2-hour weekly sessions. The intended group size was seven participants. The first session explained about MBSR and its role in promoting health. The other sessions were learning and practising stretching exercises and breathing exercises designed to reduce stress. Each group received their intervention at a location near to their home. Treatment as usual consisted of attendance at clinics and telephone support by the pulmonary hypertension nurses or doctors.

### Test data collection and patient-centred outcomes

Outcomes included physiological measures and self-report questionnaire assessment at baseline, 10–12 weeks and 6- and 15-month post-randomisation. These timings were chosen to provide long-term outcome data but also to coincide with usual outpatient clinics to avoid additional burden for participants. We conducted semi-structured interviews after the 15-month time-point with participants from both arms of the trial to determine the acceptability of participation and elicit perceptions of the MBSR the intervention from participants in this group.

We did not specify a primary outcome for the pilot trial; outcomes measured included the following:

1. Symptoms of anxiety and depression—measured respectively by the Beck Anxiety Index (BAI) and Beck Depression Index (BDI) [23, 24].
2. Measures of physical functioning—as assessed by echocardiogram, electrocardiogram, New York Heart Association (NYHA) functional class and 6-min walk test
3. Measure of health and social care resource use.
4. Health-related quality of life—measured by the components of the SF36 questionnaire [25].

The study was not powered to identify clinically important differences between MBSR and treatment as usual but aimed to determine response rates and whether a future trial would be possible.

Self-report questionnaires included a demographic sheet recording patients’ age, gender, ethnic origin and work status; BAI [23]; BDI [24]; SF-36 [25] and a patient-completed resource use questionnaire (RUQ): asking patients about their use of secondary, primary and community health care. The SF36 measures health-related quality of life on eight dimensions and two summary scores: physical and mental health [25]. All SF36 measures used norm-based scoring with population means of 50. A subset of SF-36 questions can also be used to estimate a single preference-based utility score anchored at 1 (best health) and 0 (health state as bad as death) [25, 26]. It is appreciated that the PCS and MCS components are validated against an American population and would need to be validated for this sick population before being suitable for interpretation if a full trial was to follow.

Physiological measures were collected as part of the routine clinical appointment by nursing and medical and technical staff as usual. Such physiological outcomes (e.g. echocardiogram, electrocardiogram, functional class, 6-min walk test) were collected but are not reported in this paper, which discusses the feasibility of a randomised controlled trial (RCT).

#### Session record forms

Therapists delivering MBSR completed session record forms that included staff grade and time to prepare, travel to and deliver the sessions, assistant’s time if required, equipment and materials used during the session, duration of the session, number of patients expected to attend and actually attending the session and room hire costs.

#### Blinding and measures taken to avoid bias

The PAH specialist clinical team was blinded to the treatment arm, and the statistical analysis was intended to be conducted without knowledge of patient allocation (except for health resource utilisation). Compliance with the intervention was recorded by attendance at the intervention and a diary for the set home tasks. Participants were withdrawn from the intervention if their care team identified that they were too ill to continue, or if the participant opted to discontinue attendance at sessions.

### Descriptive data and outcomes

The format and analysis for this study followed the extension of the CONSORT principles for pilot trials [27]. Participant data are presented as appropriate for a pilot study, giving numbers of those patients identified, contacted, indicating potential interest, subsequently found to be ineligible, recruited and those completing outcome measures at each stage. Descriptive statistics are presented on the outcome measures. Formal comparative analyses between arms were not undertaken.

Data from the semi-structured interviews were transcribed verbatim and analysed using inductive content analysis techniques. Words, phrases, poignant passages or concepts were assigned codes, grouped together and reduced to key themes and sub-categories to represent a holistic interpretation of the patient experience.

### Valuing and analysing resources required to deliver the intervention

MBSR was delivered by a clinical psychologist (band 8a), with assistance from a hospital clinical support worker (band 2), and valued using unit costs for health and social care tariffs [28]. The therapist delivering MBSR in London was privately hired and charged a set fee for session delivery. We used local estimates for room hire costs and recorded actual expenses for therapy materials (e.g. yoga mats, blankets, blocks and meditation bells).

The intervention was delivered in different settings for each trial centre. We assumed an opportunity cost of zero for Bristol where a hospital room was available to deliver the MBSR sessions. In Cardiff, the intervention was delivered at a local community centre at discounted room hire costs. London used a conference centre with a discounted room hire cost.

## Results

### Participants

**Fig. 1:**
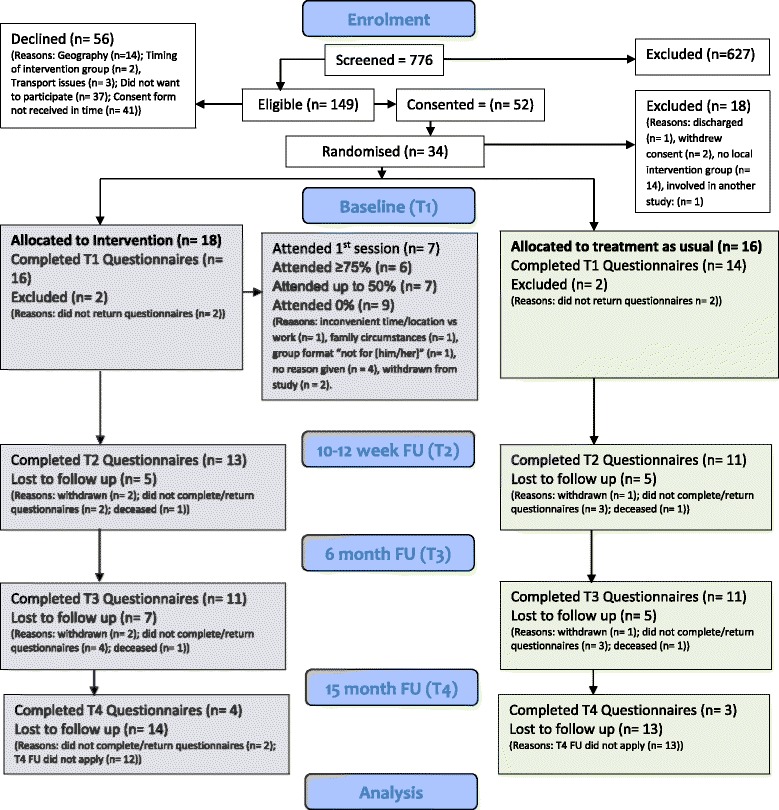
Consort diagram

Of the 181 patients attending the Bristol clinic, 71 were eligible (Fig. 1). Reasons for ineligibility included learning difficulties, young age and absence of PAH. Of the 71, 36 declined participation: 12 due to living too far away, 1 could not manage the time of the interventions, 2 had transport issues and 21 declined for other reasons. Eighteen of the 35 consented were randomised. Of those not randomised, 14 were waiting for a large enough group size in their area, 2 withdrew whilst waiting for randomisation and 1 was discharged to another clinic whilst waiting to be randomised. Four patients with pulmonary hypertension following the Fontan operation were included since their symptoms and treatment are very similar to those with pulmonary arterial hypertension. This operation is performed to palliate those patients with only one functioning ventricle.

### Acceptability of the intervention

Of the 1343 patients attending the clinic at the second recruitment centre, Hammersmith, London, 748 patients were considered ineligible as they lived too far away to travel to a weekly intervention. This left 595 potentially eligible patients for screening. Reasons for ineligibility included new patients not yet seen in clinic (*n* = 5), too young (*n* = 26), learning difficulties (*n* = 7), did not have PAH (*n* = 203), died before study commenced (*n* = 5), failed to attend (*n* = 1), poor English (*n* = 35), no reason offered (*n* = 185) and discharged before the study began (*n* = 58).

The consent forms for the remaining 78 patients were posted, of which 41 were not returned; 20 declined due to geographical location (*n* = 2), the time of the interventions (*n* = 1) and transport issues (*n* = 1); and 16 declined to take part. Hence, 17 were randomised, but 1 was excluded after randomisation as they were already enrolled in another trial.

### Feasibility of data collection

The feasibility outcomes are described in Table 1.

**Table 1:** Feasibility outcomes

Trial feasibility measure—proportion of…	Percentage (*n*/total *N*)	95% confidence interval
Patients screened as eligible (Bristol)	39.2% (71/181)	[32.1, 46.7]
Patients screened as eligible (London)	13.1% (78/595)	[10.5, 16.1]
Eligible patients consented	34.9% (52/149)	[27.2, 43.1]
Consented participants randomised	65.4% (34/52)	[50.9, 78.0]
Randomised participants with baseline questionnaire data	91.2% (31/34)	[76.3, 98.1]
Randomised participants with 3-month questionnaires	67.6% (23/34)	[49.5, 82.6]
Surviving randomised participants with 6-month questionnaires	55.9% (19/34)	[37.9, 72.8]
Group 1 participants with 15-month questionnaires	55.5% (5/9)	[21.2, 86.3]
Participants with baseline, 3- and 6-month questionnaire data	55.9% (19/34)	[37.9, 72.8]

All those consented to take part and who filled in questionnaires had baseline physical assessment data. One consented and randomised patient died after consent before data could be collected.

Of those 52 consented, only 34 were randomised to groups. This was because it was not possible to find enough patients in a certain geographical area to make group intervention possible.

Only 19 of 34 randomised completed all the questionnaires, but 45% (24/52) of patients initially recruited completed questionnaires at the 6 months follow-up. Comparison of baseline characteristics (Table 2) between those who were and were not randomised suggest that those who were randomised might be more likely to be female, not working, to have retired due to ill health, practice yoga and from the London site. The purpose of the table is therefore to show the characteristics between those who were randomised and those who were not, rather than to show outcome results, this not being the purpose of the study.

**Table 2:** Baseline characteristics and health-related quality of life comparison between those recruited and randomised and those recruited but not randomised (percentages, absolute ratios and mean and standard deviations)

Variable	Randomised	Not randomised	Total sample
Sex (% male)	20.5% (7/34)	52.9% (9/17)	31.4% (16/52)
Age (mean (min, max))	50.0 (22, 76)	53.7 (20, 88)	51.3 (20, 88)
Ethnic origin (% white British)	87.1% (27/31)	100% (17/17)	91.7% (44/48)
Currently working	32.3% (10/31)	41.2% (7/17)	35.4% (17/48)
Stopped working due to ill health?	32.2% (10/31)	23.5% (4/17)	29.1% (14/48)
Retired	31.0% (9/29)	35.3% (6/17)	32.6% (15/46)
Practice yoga regularly	9.7% (3/31)	0% (0/17)	6.3% (2/48)
Regularly meditate	9.7% (3/31)	5.9% (1/17)	8.3% (4/48)
From the primary clinic	27.3% (9/34)	100% (18/18)	51.9% (27/52)
SF-36 PCS	37.9 (11.8)	37.0 (12.0)	37.6 (11.8)
SF-36 MCS	43.4 (11.7)	48.8 (7.9)	45.3 (10.7)
SF-6D	0.632 (0.131)	0.639 (0.099)	0.634 (0.119)
Anxiety (BAI)	14.5 (11.6)	10.5 (7.3)	13.1 (10.3)
Depression (BDI)	17.3 (12.6)	10.7 (7.0)	15.1 (11.4)

The higher scores in BAI and BDI imply increased reported symptoms. BAI range was from 6 to 42, and for BDI, the range was 2–27 across both groups. The individual scores were deliberately not reported so as to avoid unintentional interpretation. There was no cut-off in the values

The protocol was found to be acceptable to many of the participants. However, as seen in the consort diagram, there was a steady attrition rate, partly due to ill health, such that only 55% were able to complete the MBSR programme. Blinding of research analysts was achieved. The fact that completion of outcome measures was incomplete might be due to the fact that patients who have limited physical mobility found it difficult to attend sessions.

Table 3 demonstrates the completeness of the data in order to inform a possible future trial using the same methodology. It is of note that the denominator at T4 is only 7 since the patients randomised at the London centre were not followed long enough to achieve this time point.

**Table 3:** The number (percentage) of randomised patients with usable data at the four time points

Variable	T1 (baseline)	T2 (2–4 weeks)	T3 (6 months)	T4 (15 months)
Date of diagnosis	17 (50%)	23 (67.65%)	18 (52.94%)	7 (77.78%)
Diagnosis	32 (94.12%)	25 (73.53%)	21 (61.76%)	7 (77.78%)
Life expectancy from now	15 (44.12%)	8 (23.53%)	9 (26.47%)	1 (11.11%)
Date of last clinic visit	26 (76.47%)	23 (67.65%)	21 (61.76%)	6 (66.67%)
Current medication	32 (94.12%)	25 (73.53%)	21 (61.76%)	7 (77.78%)
Change in symptoms	31 (91.18%)	23 (67.65%)	16 (47.06%)	6 (66.67%)
What NYHA functional class is the patient now in	32 (94.12%)	24 (70.59%)	20 (58.82%)	7 (77.78%)
Number who changed to functional class since last visit	29 (85.29%)	23 (67.65%)	19 (55.88%)	6 (66.67%)
Patient’s medication has changed since last visit?	29 (85.29%)	25 (73.53%)	20 (58.82%)	7 (77.78%)
Change given (of those who changed medication)	4 (100%)	8 (88.89%)	9 (100%)	
Reasons given (of those who changed medication)	3 (75%)	8 (88.89%)	8 (88.89%)	
Opinion of whether patient is better, the same or worse	21 (61.76%)	19 (55.88%)	16 (47.06%)	4 (44.44%)
6-min walk	19 (55.8%)	12 (35.3%)	14 (41.1%)	4
Echocardiogram	19 (55.8%)	15 (44.1%)	13 (38.2%)	3
ECG	16 (47.1%)	14 (41.1%)	15 (44.1%)	4
SF-36 score				
SF-36 PCS	91.2% (31/34)	67.6% (23/34)	52.9% (18/34)	77.8% (7/9)
SF-36 MCS	91.2% (31/34)	67.6% (23/34)	55.9% (19/34)	77.8% (7/9)
SF-6D	88.2% (30/34)	67.6% (23/34)	52.9% (18/34)	66.7% (6/9)
BAI	85.3% (29/34)	67.6% (23/34)	61.9% (21/34)	77.8% (7/9)
BDI	91.2% (31/34)	67.6% (23/34)	58.8% (20/34)	77.8% (7/9)

**Fig. 2:**
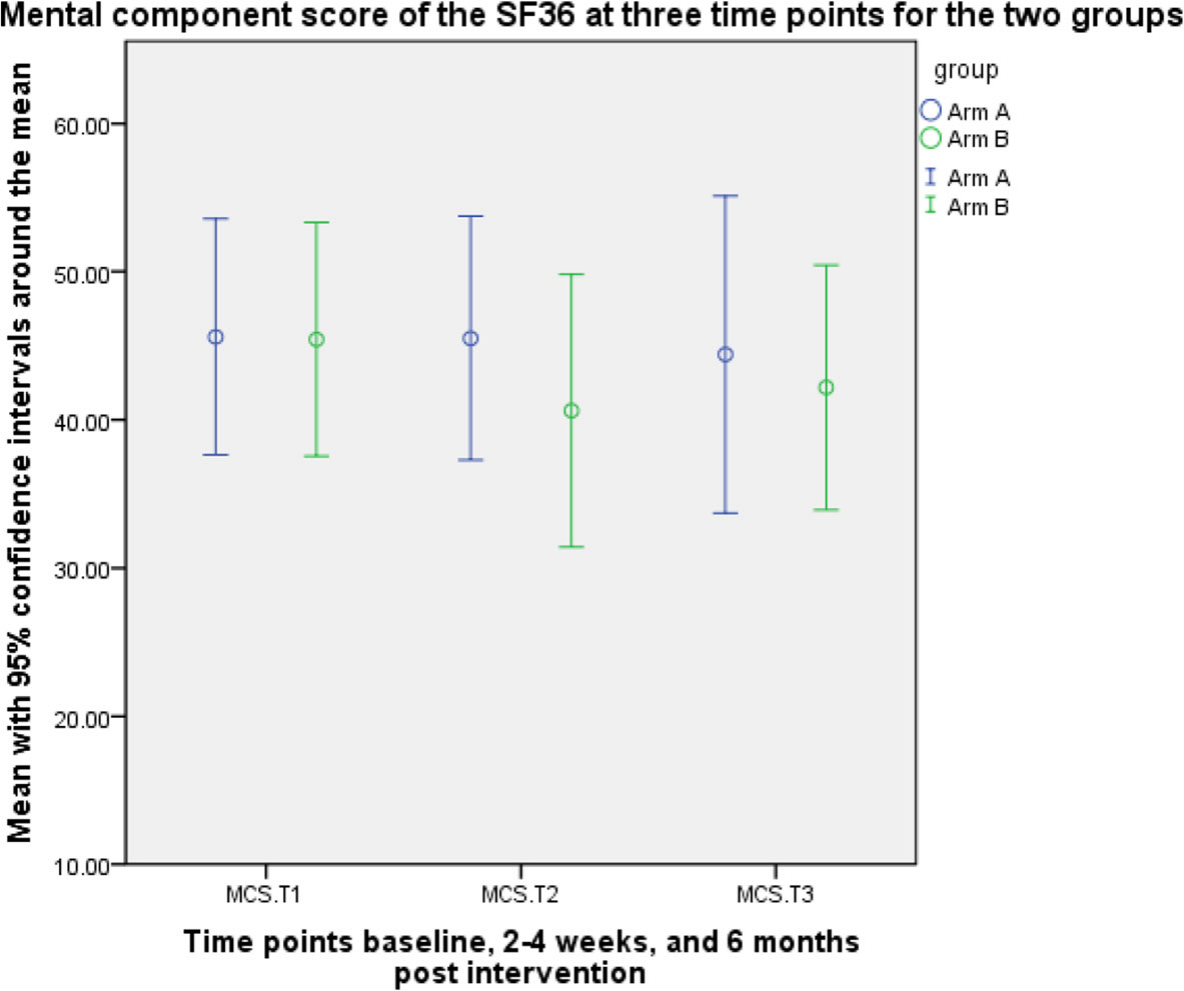
Graphical representation of the outcome data for mental component score of SF36 (time points 1 to 3 only, and only participants with data at all three time points)

**Fig. 3:**
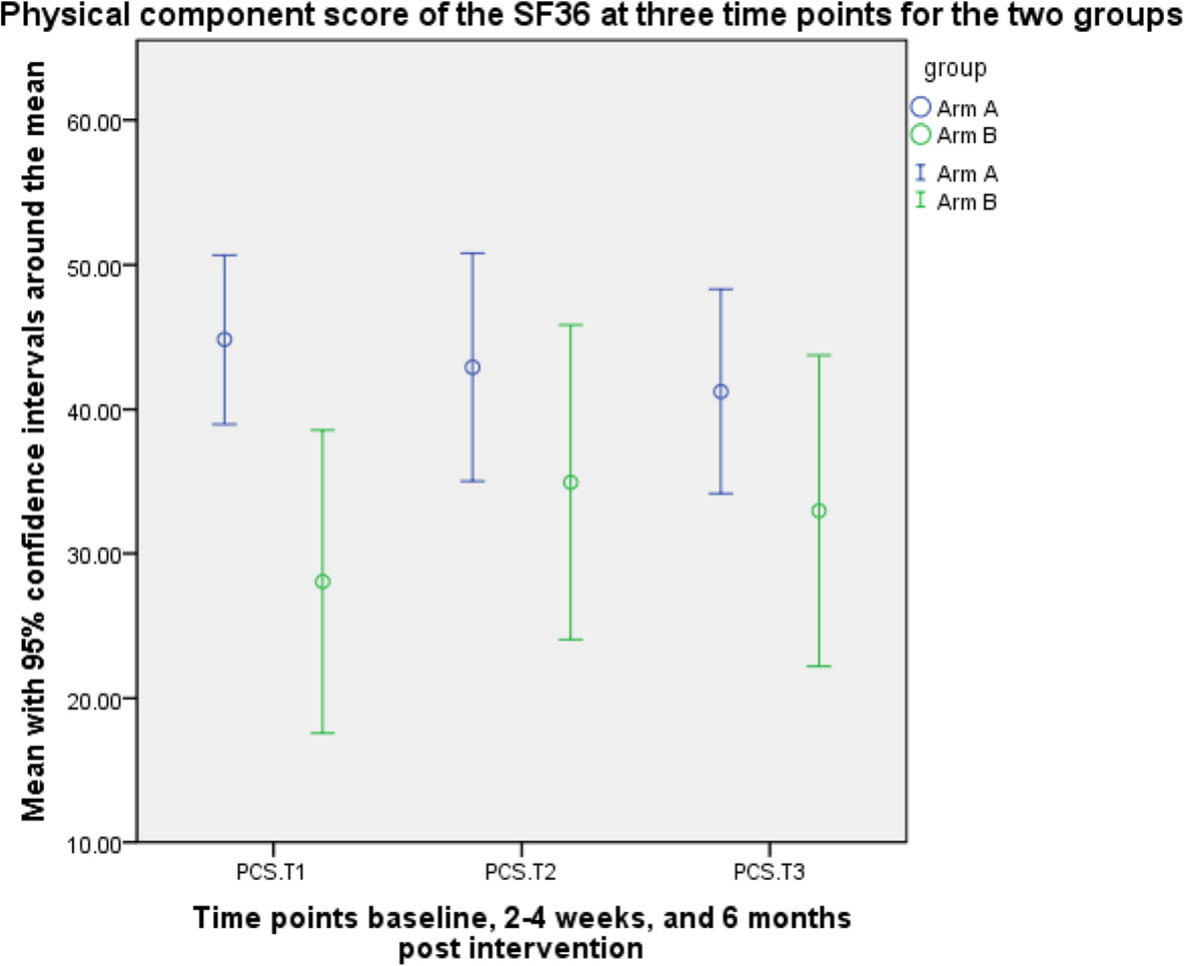
Graphical representation of the outcome data for the physical component score of the SF36 (time points 1 to 3 only, and only participants with data at all three time points)

Figures 2 and 3 demonstrate that we were able to show different components of the SF36, based on the data that we received. The labelling has deliberately been left obscure (as arm A and arm B) since the study was not powered to determine a difference between the two arms of intervention or treatment as usual. It was important that neither ourselves nor others were inadvertently trying to determine an effectiveness of the intervention since this was not the aim of the study.

### Feasibility outcomes, patient-centred outcomes and descriptive measures

The outcomes are described in Table 1.

Feasibility testing was completed by the four patients who reported finding the MBSR course highly beneficial: ‘I go out a lot more since the Mindfulness course and I am significantly more involved in what I can contribute to our day to day lives rather than sitting on the side lines, both physically and mentally. Mindfulness has moved me from being defensive and on the back foot most of the time to being far more positive to demands that are made of me and in many ways more pro-active. In a very real sense Mindfulness together with the medication is allowing me to reoccupy areas of my life I have retreated from and take on new things.’

In addition, we undertook 13 semi-structured interviews (control and five intervention patients) that gave us feedback on the acceptability of the protocol. Those who did attend the MBSR sessions found them useful, interesting and helpful in managing their symptoms and minimising the anxiety and psychosomatic symptoms. This has informed us of several key themes: (1) wanting to help, (2) challenges with questionnaire completion, (3) preferred time of year and (4) taking part again.

#### Theme 1: Wanting to help

Six participants identified a sense of being indebted, wanting to help or ‘give something back’.

Truly, totally wonderful people there… They just say ‘would you like to take part in a survey’ and I just say ‘yes I will’ ….. I had no idea what on earth I was signing up for. 1119, female, age 69.

I was asked if I wanted to do it and I’m one of those people…where they always ask me - yeah, alright, you’ve got me, use me type of thing. So I was like yeah, I don’t mind. 1123, female, age 53.

I was happy to go ahead if it helps. 1026, male, age 39.

#### Theme 2: Challenges with questionnaire completion

Many found the psychological questionnaires quite long and repetitive, which possibly influenced how individuals approached answering them.

There seemed to be quite a lot of questions although fairly quick to complete*.* 1112, female, age 55.

I thought some of them were a bit long and maybe very repetitive….It didn’t take too long – about half an hour to three quarters of an hour. 1123, female, age 53.

I found the questionnaires quite straightforward. There was one of the questionnaires that I always found a little bit difficult from the point of view that the questions were very leading….There was no room for manoeuvre,,, it was either sort of trying to draw you to the conclusion that you were quite depressed or not at all depressed, there was no middle road…..so it felt a little bit, sort of contrived almost. 1011, male, age 43.

#### Theme 3: Preferred time of year and time of day of intervention

In the winter, the darker evening and cold weather were seen as quite detrimental to participation, but there were varied opinions around the time of day.

I mean, I understand it had to be that time of day but the thing was it was winter so it was dark ….. and wet and cold…. I think that would have put people off. If it had been earlier in the day or in the Spring or Summer, I think you would’ve got a lot more people to go. 1070, female, age 41.

I think, although I had to get up earlier, it was a good time of day to get up because it means you have the rest of the day. 1127, female, age 63.

#### Theme 4: Taking part again

Seven of the 13 interviewees expressed a willingness to take part either in the study as a whole or the intervention again if asked.

Yes, I don’t mind doing it again if it helps. 1079, male, age 68.

It was a good experience and I wish I could go regularly. 1070, female, age 41.

I quite enjoyed it and I would do it again if you asked me. 1125, female, age 61.

Nothing really worried me one way or the other. I’d do it again. 1026, male, age 39.

Participants report that they found the MBSR techniques helpful in assisting with management of their symptoms and have continued to use them many months after the study period finished.

### Cost of delivering MBSR

The average cost of delivering the eight MBSR sessions was similar in Bristol and Cardiff, £2234 (SD £286) and £2423 (SD £295), respectively, but higher in London £4128 (SD £270) (Table 4). Higher costs in London reflect the private therapist fee and higher room hire costs (between £160 and £260 per room, compared to £15 per room at the local community centre in Cardiff).

**Table 4:** Number of patients randomised and attending the MBSR intervention and costs of delivery of eight MBSR sessions per centre, in total, per patient expected and per patient attending

	Number of patients randomised to attend the sessions	Number of patients on average attending the sessions	Mean total cost	SD	Mean cost per patient expected to attend sessions	SD	Mean cost per patient attending the sessions	SD
Bristol	6	1.75	£2234	£286	£372	£48	£1443	£504
Cardiff	5	2	£2423	£295	£485	£59	£1211	£148
London	7	2.75	£4128	£270	£590	£39	£1538	£264

The cost of the intervention is highly sensitive to the number of patients attending the sessions. Although we invited seven patients to attend each group, actual attendance averaged one to two (Bristol), two (Cardiff) and two to three (London) patients. Therefore, the average cost per patient attending MBSR in the trial was high and ranged from £1211 in Cardiff to £1538 in London.

## Discussion

This study provides insight into the difficulty of recruiting to a group-based intervention study in a rare disease. Many of the patients found the therapy helpful, but it is not yet possible to determine its benefit. There are significant barriers to implementing face-to-face group therapies in rare diseases with geographically dispersed patients. Research on these interventions is difficult as numbers are constrained. For those who were clinically stable and had little limitation, demands of professional employment often prevented travel to intervention during working hours. For those who were unwell, it was too much of an imposition to travel to the sessions, especially in the winter months.

The work of Bauer-Wu has attempted to address this concern that sick patients may be too unwell to travel to group mindfulness sessions. Bauer-Wu undertook one-on-one sessions for 17 min on a daily basis with those undergoing stem cell transplantation. 78.9% completed the interventions, considerably more than in the present study [29]. Others have tried undertaking the MBSR programmes over six telephone conversations [30] or even undertaking the whole programme in a single session, based on the acceptance and commitment model [31]. These models show that there may be other methods of delivering mindfulness, although the validation of their effectiveness is still rather lacking in the longer term, perhaps requiring increased re-enforcement. In light of the chronic ill health of our patients with PAH and the fact that it is a relatively rare disease, such alternatives might offer better means of delivering the intervention.

Due to the wish to recruit sufficient people for the expected size of intervention groups, we tested out the approach of topping up the group numbers with patients with similar clinical diagnoses. This was found to be a useful and feasible way of randomising at a site with low patient numbers. We would recommend this method to others attempting recruitment to group interventions. It is not clear whether the MBSR programme which is tailored for people with PAH would be less appropriate for those with similar diagnoses or whether the inclusion of people without PAH might reduce the efficacy of the tailored intervention for those with the disease.

MBSR however does appeal to a significant proportion of this patient population. The semi-structured interviews revealed that those who attended were pleasantly surprised by the effect of MBSR and continued to practise many months later. It is possible that a more successful intervention would use the same techniques remotely, via e-health devices such as a smartphone app or a web or video-based programme or combination of these. Some studies using Internet-based MBSR programmes have already been introduced [32]. This would allow the benefit for the patients without the need for travel to group meetings. Those who found the timing of the meetings restrictive due to work or other commitments would be able to take part when suitable. Achieving better MBSR attendance is very important in order to maximise the potential benefit of MBSR and make it more likely to be cost-effective for the NHS. Larger groups (e.g up to 12 patients) might be preferable but will be difficult to achieve for a rare disease like PAH.

## Conclusion

It is clear that the methodology of this pilot trial will not translate more generally to other rare diseases in its current form. However, we have devised a suitable MBSR programme, which may be of benefit in PAH, a debilitating and uncommon disease. This study highlights the potential benefits of MBSR for patients with rare diseases whilst drawing attention to the significant challenges posed in delivering robust evaluations of group-based MBSR in these populations. It is possible that future research could use the same methodology but would need to be delivered in a way to accommodate at home use as travel was the main factor which prevented participation. This might be by an “e” programme delivered online or by some other telephone or single visit method—as others have tried.

## Abbreviations

APAH: Associated pulmonary arterial hypertension
BAI: Beck Anxiety Index
BDI: Beck Depression Index
MBSR: Mindfulness-based stress reduction
NYHA: New York Heart Association
PAH: Pulmonary arterial hypertension
RCT: Randomised controlled trial
RUQ: Resource use questionnaire
SF36: Health-related quality of life index
